# A Concurrent Cognitive Task Does Not Perturb Quiet Standing in Fibromyalgia and Chronic Fatigue Syndrome

**DOI:** 10.1155/2018/9014232

**Published:** 2018-08-07

**Authors:** Omid Rasouli, Egil A. Fors, Ottar Vasseljen, Ann-Katrin Stensdotter

**Affiliations:** ^1^Department of Neuromedicine and Movement Science, Faculty of Medicine and Health Sciences, Norwegian University of Science and Technology (NTNU), Trondheim, Norway; ^2^Department of Public Health and Nursing, Faculty of Medicine and Health Sciences, Norwegian University of Science and Technology (NTNU), Trondheim, Norway

## Abstract

**Background and Objectives:**

Cognitive complaints are common in fibromyalgia (FM) and chronic fatigue syndrome (CFS). Fatigue as well as pain may require greater effort to perform cognitive tasks, thereby increasing the load on processing in the central nervous system and interfering with motor control.

**Methods:**

The effect of a concurrent arithmetic cognitive task on postural control during quiet standing was investigated in 75 women (aged 19–49 years) and compared between FM, CFS, and matched controls (*n*=25/group). Quiet standing on a force plate was performed for 60 s/condition, with and without a concurrent cognitive task. The center of pressure data was decomposed into a slow component and a fast component representing postural sway and adjusting ankle torque.

**Results:**

Compared to controls, CFS and FM displayed lower frequency in the slow component (*p* < 0.001), and CFS displayed greater amplitude in the slow (*p*=0.038 and *p*=0.018) and fast (*p*=0.045) components. There were no interactions indicating different responses to the added cognitive task between any of the three groups.

**Conclusion:**

Patients displayed insufficient postural control across both conditions, while the concurrent cognitive task did not perturb quiet standing. Fatigue but not pain correlated with postural control variables.

## 1. Introduction

Executive function generally requires concerted cognitive and motor ability. In patients suffering pain and fatigue, there is evidence of cognitive difficulties as well as motor control deficits [1, 2], and patients often complain about increased effort and difficulty performing cognitive tasks [3, 4]. Cognitive dysfunctions, including working memory impairment, attention deficit, and less efficient information-processing capacity, are core symptoms reporting pain and fatigue conditions, specifically in chronic fatigue syndrome (CFS) and in fibromyalgia (FM). These patients are often more troubled by cognitive difficulties than by other symptoms [5]. Neuroimaging has demonstrated functional and structural alterations in the central nervous system (CNS) and a pattern of increased neural recruitment during cognitive tasks in both CFS [6] and FM [7]. Increased effort in these patients to perform is reported after physical as well as mental exertion, and is seen as lingering postexertional fatigue [8, 9]. Whether a greater mental effort and increased neural recruitment during cognitive tasks may interfere with motor control remains to be shown.

Possible consequences of pain and fatigue on motor function are often forgotten but need to be recognized. Cognitive neuroscience has shown that pain and fatigue are overlapping symptoms between different conditions and diagnoses and may be an effect of chronification with changes in similar regulatory mechanisms in the central nervous system (CNS), particularly in domains not under voluntary control [10]. This may affect certain aspects of motor performance, as sensory motor learning requires ability to maintain and update internal models. This is a prerequisite for prediction of action, necessary to allow a series of events to be contained without the need of voluntary control [11]. Perception of pain and fatigue may interfere with this process as deficits in balance and postural steadiness have been demonstrated in patients with FM as well as in CFS [12, 13] with similar deficits found in dynamic postural control at gait initiation [14].

Notably, brain-body-environment interactions and perception-action links are common basis for behavior without representational separation between domains [15]. Deficits in either domain may thus affect performance when executed concurrently due to sharing of neural networks between cortical areas [16]. Attention and sensory integration are essential to produce appropriate motor output such as balance control [17]. In some populations, particularly the fragile elderly, the risk of falling increases with the addition of a concurrent task such as talking while walking [18], and execution of multiple tasks is a major risk factor for falls [19]. Notably, there is some evidence suggesting premature aging of brain areas in both FM and CFS [20, 21], which may affect the execution of multiple tasks in persons with these diagnoses.

The addition of a cognitive task while maintaining postural control in quiet standing is thus expected to increase the load on central processing and therefore affects the ability to sustain postural equilibrium. In healthy persons, the controlling strategy appears to tighten to maintain postural equilibrium when a cognitive component is added. As an effect, postural sway may decrease [22]. In contrast, elderly with risk of falling display increased rather than decreased postural sway [23].

A dual task paradigm is generally used to study the interference between postural control and cognitive loading [17], and was therefore considered appropriate to investigate motor responses during quiet standing in FM and CFS. To find whether patients with FM and CFS would respond either similarly to healthy young individuals with reduced postural sway [24] or similar to elderly with increased postural sway [23] and greater regulatory force [25], both the controlled and the controlling parameters need to be assessed [26]. That is, on the level of performance and on the level of control of posture. A means to asses this is via structural data analyses by decomposition of ground reaction forces registered by a force platform. In addition to measures in the time domain that describe the magnitude of these components, additional measurements in the frequency domain are necessary to define the control strategies [27, 28]. Impaired postural control and the effect of added cost of a concurrent task may be expressed as deviations in both components in both the time and frequency domains.

Given the evidence that pain as well as fatigue affects motor and cognitive ability and findings of reduced postural stability, cognitive complaints, problems with sensory integration, and signs of premature aging in patients with FM and in patients with CFS, it was hypothesized that a cognitive task would cause an increased load on central processing in both patient groups. This would cause reduced drive for maintaining satisfactory postural control. Hence, postural sway was expected to increase, similar to in elderly [25].

## 2. Methods

A cross-sectional case-control study was designed to investigate the effect of a concurrent cognitive task on postural control parameters during quiet standing. Seventy-five females, aged 19–49 years, participated in this study (Table 1). The number of participants was estimated based on a previous study, using a similar protocol, on schizophrenic patients with 30 participants divided into two groups [29]. In the present study with three groups and less severe conditions, the number of participants was increased to 25 in each group. Inclusion criteria were young to middle aged females, as diagnoses of CFS and FM are predominant in women within this age span. Patients diagnosed with both FM and CFS were excluded. Eighty-seven patients were found eligible for participation, and those interested were referred by their attending physician and included consecutively during a period of 20 months. Thirty-two declined and data from four were excluded due to unsatisfactory quality. One patient with FM did not complete the test due to pain. Data were used from 25 patients diagnosed with CFS according to the Centers for Disease Control and Prevention criteria [30] and 25 patients diagnosed with FM according to the American College of Rheumatology (ACR) 1990 criteria [31]. Diagnoses were determined in collaboration between a rheumatologist, psychiatrist, and neurologist at the National Competence Centre for Complex Symptom Disorders. Condition severity was determined by the Fibromyalgia Impact Questionnaire (FIQ) and the Chalder Fatigue Scale. Twenty-five healthy control (HC) persons recruited from students and staff of the hospital and university constituted an age- and gender-matched control group with no history of chronic pain or fatigue. Exclusion criteria for all were diagnosed psychiatric disorder, clinical depression, neurological condition, musculoskeletal disorder, vestibular deficits, or uncorrected reduced vision potentially interfering with postural control. Verbal and written information was given, and written informed consent was obtained from each participant. The study was registered in Clinical Trials (NCT01686074) and approved by the Regional Ethical Committee for Medical and Health Research Ethics (2012/679/REK midt) and conducted according to the Declaration of Helsinki.

### 2.1. Data Acquisition

Participants performed two different trials of quiet standing with open eyes on a firm surface: a baseline condition without a concurrent cognitive task and an experimental condition with a concurrent cognitive task. The concurrent task consisted of counting backward aloud from 150 in steps of 7, designed to ensure actual execution of the cognitive task and minimize verbalization to prevent rhythmic counting and breathing, potentially influencing quiet standing performance [32]. Three-dimensional (3D) ground reaction forces were registered at 100 Hz with a Kistler force plate (9260AA6; Kistler Instruments AG, Switzerland).

Each test was performed once, for 60 seconds [33], without shoes, feet parallel, and arms folded across the chest. Feet width was individually standardized as the distance equal to half the shoulder width between the acromial processes and marked on the platform to ensure the same foot position for both conditions. All participants started with the baseline condition to ensure equal potential learning and/or fatigue effect. Instructions were to step onto the platform, stand still and relaxed without moving the head or extremities, or talk except articulating the numbers. A red cross (21 × 21 cm), 4 m away at eye level, served as a visual reference point for postural control. To establish a steady, quiet stance, the participant was informed that the test was commenced 10 s before the recording started and that it was finished 3 s after the data collection was completed. One-minute seated rest was provided between the conditions.

### 2.2. Data Analysis

Fifty-eight seconds of data were used from each trial, excluding one second at the start and end of the recording to avoid potential electronic noise from the start and stop key. Data were analyzed in MATLAB (R2014a; MathWorks Inc., Natick, MA). The preprocessed center of pressure (CoP) signals (2nd order Butterworth, 8 Hz, low pass, zero lag) was decomposed into a slow component and a fast component according to the concept of instant equilibrium forces [26]. Instants of equilibrium points in the signal, when the total horizontal force equals zero, were identified, and the CoP positions at these instants were determined and interpolated by a cubic spline function for estimation of the trajectory of the slow component. The slow component is ascribed to the movement of the center of mass (CoM) of the body, that is, postural sway. Deviation of CoP from the approximated trajectory of the slow component was determined as an estimation of the trajectory of the fast component. The fast component can be attributed to the torque created by the movement of the ankle joint to control postural sway [26].

Amplitude and frequency parameters were computed for the slow and fast components. Amplitudes were calculated by computing the 95% confidence ellipse area (mm^2^), defined from the first two principal components of each of the slow and fast components [34]. The two radii of the ellipses were defined by the mediolateral (ML) and anteroposterior (AP) directions. The frequency content of the slow and fast components in the ML and AP directions were estimated by the Fourier analysis of the characteristics of the power spectral density using Welch's periodogram method [34].

### 2.3. Statistical Analysis

The statistics were performed using the SPSS statistical software (Version 22; IBM Corporation, USA). Normal distribution was verified with P-P plots, and histograms were inspected for control of skewness and kurtosis. A mixed-design analysis of variance (ANOVA) was used to analyze the effect of group, condition, and interaction (group × condition), with group (*n*=3; HC, CFS, and FM) as the between-subjects factor and condition (*n*=2; baseline and experimental) as the within-subjects factor. Sphericity was determined according to Mauchly's test. Wilks' lambda was used for multivariate exact statistics for between-subjects effects. Pairwise comparisons with Bonferroni corrections were performed to identify significant differences between groups and between conditions. Pearson correlations were used to investigate the influence of pain, fatigue, and education on postural control variables in the patient groups. Age, weight, and BMI did not differ between the three groups (Table 1), and there were no correlations between these variables and the outcome variables. Therefore, they were not included as covariates. Partial eta-squared (*η*^2^*p*) was used for effect size. The alpha level was set at *p* < 0.05.

## 3. Results

**Figure 1:**
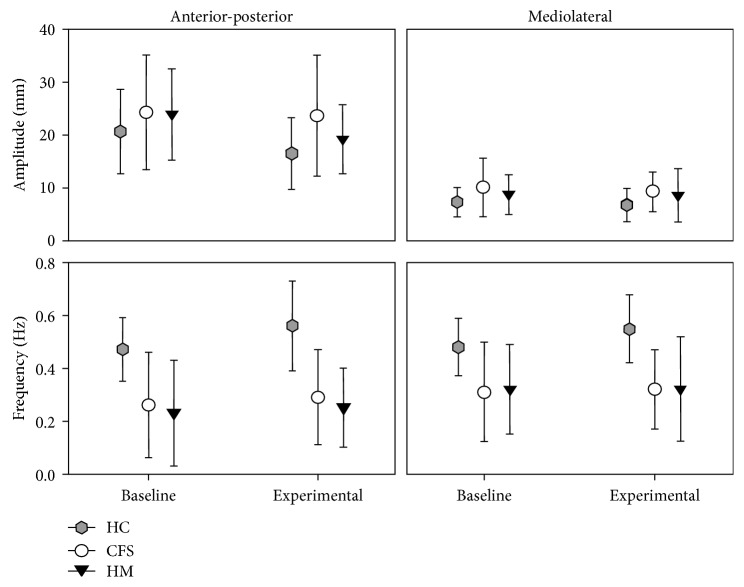
Estimated group means (standard error) of the slow component derived from the center of pressure data representing postural sway during quiet standing in both anteroposterior and mediolateral directions. Baseline and experimental conditions with the concurrent cognitive task are shown.

**Figure 2:**
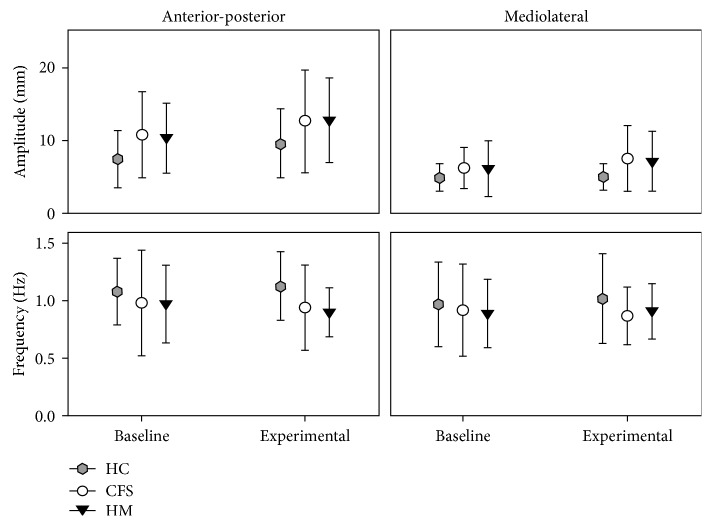
Estimated group means (standard error) of the fast component derived from the center of pressure data representing adjusting ankle torque during quiet standing in both directions. Baseline and experimental conditions with the concurrent cognitive task are shown.

Overall, there were no statistical differences between CFS and FM, and both groups displayed generally similar and greater amplitudes and lower frequencies for the slow component as well as the fast component compared to HC, across both conditions (Figures 1 and 2; Table 2). Differences in strategy for postural control as an effect of the added concurrent cognitive task could not be inferred from single variables as there were no significant interactions between group and condition on any variable. Table 2 shows the results of the main effects and post hoc comparisons. Across patient groups, there were some scattered correlations between fatigue variables with postural control, but no correlations with pain variables.

### 3.1. The Slow Component

The analysis showed that *amplitude (AP and ML)* differed between groups: *F*_2;71_=3.96, *p*=0.023, *η*^2^*p*=0.100 (Figure 1). Significant main effects of the group were found for amplitude in both the AP (*p*=0.044) and the ML (*p*=0.020) directions. Post hoc analyses showed greater amplitude for CFS compared to HC in the AP (*p*=0.038) and the ML (*p*=0.018) directions, but no differences were found between FM and HC (Figure 1; Table 2).

The analysis showed that *frequency* differed between groups: *F*_2;71_=7.03, *p*=0.002, *η*^2^*p*=0.162 (Figure 1). Significant main effects of the group were found for frequency in both AP (*p* < 0.001) and ML (*p* < 0.001) directions. Post hoc comparisons showed similar and lower frequency for both FM and CFS compared to HC in both the AP and the ML directions (*p* < 0.001 for all) (Figure 1; Table 2).

As an effect of condition, the amplitude in the AP direction decreased (*p*=0.006) with the experimental task, whereas the frequency increased in both the AP (*p*=0.02) and the ML (*p*=0.030) directions.

### 3.2. The Fast Component

The analysis showed that *amplitude (AP and ML)* differed between groups: *F*_2;71_=4.38, *p*=0.016, *η*^2^*p*=0.110 (Figure 2). There was a significant main effect of the group for amplitude in the AP (*p*=0.033) and the ML (*p*=0.031) directions. Post hoc analysis showed a greater amplitude only in the ML direction and only for CFS (*p*=0.045) compared to HC (Figure 2; Table 2). A multivariate analysis for the AP and ML directions showed that *frequency* did not differ between groups: *F*_2;71_=0.20, *p*=0.820, *η*^2^*p*=0.006.

As an effect of condition, the amplitude increased only in the AP direction (*p*=0.005) as a response to the experimental task, whereas the frequency did not change significantly (Figure 2; Table 2).

### 3.3. Correlations

Across patient groups, the frequency in the AP direction in the slow component increased with fatigue before the test across both conditions (baseline *r*=0.306, *p*=0.030; experimental *r*=0.329, *p*=0.020). In the experimental condition, the frequency in the AP direction in the slow component also increased with fatigue after the test (*r*=0.332, *p*=0.019) and with Chalder's fatigue score (*r*=0.350, *p*=0.001). In the experimental condition, Chalder's fatigue score also correlated positively with the frequency in the ML direction in the slow component (*r*=0.318, *p*=0.024). At baseline, the amplitude in the AP direction in the fast component correlated with fatigue before the test (baseline *r*=0.405, *p*=0.003). Pain variables, including FIQ, and education did not correlate with postural control.

## 4. Discussion

This study is, to our knowledge, the first that collectively evaluated the effect of a concurrent cognitive task on postural control in quiet standing in patients with FM and CFS compared to controls. The study comprised both the fast and the slow components derived from CoP measurements, defining the controlling variable (attributed to adjusting ankle torque) and the controlled variable (ascribed to postural sway), respectively. The results supported our hypothesis only in part, showing unsatisfactory postural control in both patient groups, characterized by larger amplitudes and lower frequencies for the slow and fast component in both the anteroposterior and mediolateral directions during both conditions, with and without a concurrent cognitive task. There were no significant differences between the patient groups, but the CFS group performed in general worse than the FM group when compared to controls. We found no interactions that supported different patterns of postural control strategies in response to the added cognitive task in any of the patient groups compared to controls. As seen in Figures 1 and 2, the profiles of the responses are similar for all groups.

Both patient groups displayed larger amplitude in the slow component, which represents the movement of the body's CoM, thus indicating larger postural sway. This suggests worse performance in patients. Both patient groups also displayed larger amplitudes in the fast component, which is attributed to lateral forces that controls the position and movement of CoM. This suggests a deficit in control where the ankle torque is too large relative to the frequency. Notably, the frequency of the fast component was similar across all three groups. In theory, if the torque is too large relative to the frequency, it will cause greater postural sway as CoM is pushed too far in one direction before a counteracting force is created [29]. Alternatively, larger postural sway may be due to that the timing of the adjusting ankle torque was not sufficiently synchronized to correct the drift of CoM at the right moment. This assumption fits with the Drift-and-Act Hypothesis, which proposes that postural control includes a sequence of drift-and-act episodes where the body deviates from the vertical line until the sensory information has been processed in the CNS and a corrective action is initiated [35].

Our hypothesis that the amplitude of the slow component would increase and amplitude and frequency in the fast component decrease in patients as an effect of the concurrent cognitive task, indicating larger postural sway and reduced drive to maintain adequate postural control, was not supported. Even though patients in general displayed larger amplitudes relative to the frequency in the fast component, and larger amplitudes and lower frequencies in the slow component, the intrinsic patterns at baseline and in the experimental task followed a similar profile in all groups. This implies that the nature of the response to an added cognitive task was not different in patients compared to controls. Similar responses in healthy subjects to added cognitive loading during quiet standing were presented in previous studies, showing reduced CoP area [36] and sway amplitude [37] and increased sway frequency [32]. CoP is a measure of the migration of the total reaction force across the support surface and postural sway is the parameter that the system needs to control. In addition to measures used in these previous studies mentioned above, the present study also assessed the fast component in the CoP signal, which is interpreted as the lateral and controlling force that can be attributed to ankle torque [26]. Increased amplitude in the fast component confirmed the assumption of upgraded control with a concurrent cognitive task in quiet standing, proposed by Dault et al. [32]. This furthermore supports the assumption that arousal and postural control are related and that a concurrent cognitive task can increase arousal and attention compared to only quiet standing without an additional task, thus upgrading the control [38].

Performing a concurrent cognitive task in quiet standing increases the load on central processing [37], and was expected to negatively affect postural control in the patients. Although the amplitude of the slow component was generally larger in patients, it decreased while performing the concurrent cognitive task, which reflects a normal response to an added or increased cognitive load [37]. This normal response in patients may be explained by that the level of difficulty for either the cognitive or the postural task was too low to challenge control. Other explanations may be that patients were, despite their diagnoses, in relatively good shape and participated on a good day. We noted several cancellations due to feeling too unwell to come to the lab, and one patient who was unable to finish the test due to pain. Thus, a more challenging test may, or may not, trigger responses similar to in elderly with risk of falling, that is, larger amplitude in the slow component and reduced amplitude and/or frequency in the fast component. That the cognitive task did not cause increased amplitude in the fast component may also be explained by the “posture first principle” [39] as attention is typically aimed toward postural control at the cost of other tasks, to secure stability, provided a sufficient level of control.

The generally worse performance in patients compared to controls may be explained by several factors. Deficits in the sensory motor processing [40] and neurological symptoms such as muscle weakness and poor balance [41] have been reported in both FM and CFS. Evidence of the accelerated age-related decrease in white and gray matter in the CNS has been shown in both patient groups [20, 21], suggesting reduced ability in central processing comparable to elderly persons, including cognitive dysfunction that links to postural instability [42]. Reduced attentional and cognitive capacity as a result of pain and fatigue may contribute to explain the generally reduced postural control displayed in patients in the present study [43]. Multiple factors could potentially contribute to explain the reduced control of standing posture, more specifically, delayed triggering of a corrective action, and a slow action process [1, 44].

In the present study, we could however not find any correlations between pain, before or after the test or for FIQ, with any of the postural control variables. Fatigue before and after the test as well as the Chalder Fatigue Scale did in contrast correlate with several, but apparently scattered, postural control variables. Two correlations were found at baseline, while four were found in the experimental condition. Of those, two were for the same variable, frequency for the slow component in the AP direction, which suggests that the correlations were not totally random. It was however counter intuitive that the correlations with fatigue were positive for frequency, as frequency was generally lower in patients. Higher frequency and larger amplitude do however add up to higher velocity, and velocity is shown to be the most important cue that the system uses for control of posture rather than position or acceleration [45]. For a full explanation of the nature of postural control deficits, several different measurements may have to be considered.

Post hoc comparisons revealed larger and other differences for postural control in patients with CFS than in patients with FM when compared to controls. This finding was in line with shown correlations between fatigue and postural control. Notably, there is up to 70% diagnostic overlap between CFS and FM [46], indicating that fatigue is common also in FM. Note that education did not correlate with postural control variables in the present study. Thus, fatigue rather than pain or cognition may explain demonstrated deficits in postural control.

Although the response to the concurrent cognitive task in patients did not indicate reduced control as expected, the number of correlations between fatigue and postural control variables increased. Importantly, there is a link between mental fatigue and cognition, at least in CFS [3]. Previous studies seem to point to different cognitive deficits in these patient groups that may depend on fatigue. Slow information processing has been demonstrated in patients with CFS, while patients with FM display impaired ability of attention [5]. A review of the current research on neuropsychological functioning in CFS shows that slowed processing speed, impaired working memory, and poor retention of information are the most prominent features of cognitive dysfunction [47]. Note, however, that more recent research in cognitive neuroscience claim that overlapping symptoms between these conditions may be an effect of chronification with changes in similar regulatory mechanisms in the CNS [10]. Essentially, described discrepancies between cognitive difficulties in FM and CFS may thus owe to different study methods in different studies rather than true differences in underlying deficits. Recent studies support the findings of motor and cognitive affection both in patients with CFS [1] and FM [2], but hitherto no specific or unique patterns of cerebral changes have been found that distinguish these conditions from each other.

Future research on the effect on postural control of concurrent tasks should increase the level of task difficulty to challenge capacity in the patients. Future research should also study the interrelationship in central processing between pain and fatigue with cognition and motor control.

The present results should be interpreted with reservation to the following limitations: we did not assess the cognitive functioning, and medications were not controlled beyond the use of analgesics for pain. Verbalization and respiratory pattern, as potential interference with postural sway, was not monitored. Although the cognitive task was designed to minimize verbalization, to prevent rhythmic counting and thereby rhythmic breathing, the frequency of verbalization was not standardized or controlled for. A relatively small sample size may limit external validity due to the great heterogeneity of symptoms in both CFS and FM.

## 5. Conclusion

The intrinsic patterns at baseline and in the experimental condition followed a similar profile in all groups, without any interactions that supported different postural control strategies in response to the concurrent cognitive task in patients. Both patient groups did however display insufficient postural control compared to control persons, generally characterized by larger amplitude and lower frequency in the slow component representing postural sway, and larger amplitude in the fast component attributed to adjusting ankle torque. It is proposed that a mismatch between the magnitude and frequency of the controlling ankle torque induced greater postural sway in patients. The CFS group displayed greater differences than the FM group compared to controls, suggesting a somewhat worse general performance. Correlations between fatigue and postural control but not between pain and postural control indicate that fatigue is the explaining factor for reduced postural control in both groups. There were no statistical differences between patient groups.

## Figures and Tables

**Table 1:** Characteristics of the participants in each group.

Variables	HC (*N*=25)	CFS (*N*=25)	FM (*N*=25)
Age (years)	34.4 (7.9)	34.0 (8.9)	38.6 (8.0)
Weight (kg)	68.0 (9.8)	71.6 (12.9)	75.4 (14.3)
Height (cm)	167.2 (7.1)	169.1 (5.4)	168.5 (6.0)
BMI (kg/m^2^)	24.3 (3.5)	25.2 (5.1)	26.5 (4.5)
Education (years)	16.1 (2.3)^*∗∗*^	13.4 (2.5)^*∗∗*^	13.5 (2.2)^*∗∗*^
Pain level^a^	0.08 (0.28)^*∗*^^,^^*∗∗*^	1 (1.16)^*∗*^	3.7 (1.8)^*∗∗*^
Fatigue level^a^	0.6 (0.8)^*∗∗*^	3 (1.8)^*∗∗*^	3.2 (2.2)^*∗∗*^
Chalder score	5.8 (5.7)^*∗*^^,^^*∗∗*^	25.4 (3.8)^*∗∗*^	21.1 (5)^*∗*^
FIQ	—	—	56.9 (13)

Data are presented as means (SD). HC: healthy control; CFS: chronic fatigue syndrome; FM: fibromyalgia; FIQ: Fibromyalgia Impact Questionnaire. ^a^Level of pain and fatigue on the day of testing registered upon arrival to the lab. ^*∗*^Significance level 0.05; ^*∗∗*^significance level 0.01.

**Table 2:** The results of repeated-measures ANOVA for each variable.

	Variable	Group ^*∗*^ condition	Condition	Group	Post hoc
Area	CoP	*F*(2, 81) = 0.45	*F*(1, 81) = 0.76	*F*(2, 81) = 6.16^*∗∗*^	HC-CFS^*∗∗*^, HC-FM^*∗*^
	Postural sway	*F*(2, 81) = 0.20	*F*(1, 81) = 4.32^*∗*^	*F*(2, 81) = 5.08^*∗∗*^	HC-CFS^*∗∗*^
	Adjusting ankle torque	*F*(2, 81) = 2.41	*F*(1, 81) = 15.93^*∗∗*^	*F*(2, 81) = 5.97^*∗∗*^	HC-FM^*∗∗*^

Postural sway	Amp-AP	*F*(2, 81) = 0.84	*F*(1, 81) = 6.92^*∗*^	*F*(2, 81) = 4.36^*∗*^	HC-CFS^*∗*^
	Amp-ML	*F*(2, 81) = 0.41	*F*(1, 81) = 0.71	*F*(2, 81) = 4.90^*∗*^	HC-CFS^*∗*^
	F-AP	*F*(2, 81) = 1.61	*F*(1, 81) = 13.08^*∗∗*^	*F*(2, 81) = 22.08^*∗∗*^	HC-CFS^*∗∗*^, HC-FM^*∗∗*^
	F-ML	*F*(2, 81) = 1.24	*F*(1, 81) = 5.67^*∗*^	*F*(2, 81) = 16.01^*∗∗*^	HC-CFS^*∗∗*^, HC-FM^*∗∗*^

Adjusting ankle torque	Amp-AP	*F*(2, 81) = 1.07	*F*(1, 81) = 12.38^*∗∗*^	*F*(2, 81) = 4.89^*∗*^	HC-FM^*∗*^
	Amp-ML	*F*(2, 81) = 0.70	*F*(1, 81) = 4.79^*∗*^	*F*(2, 81) = 4.99^*∗∗*^	HC-CFS^*∗*^, HC-FM^*∗*^
	F-AP	*F*(2, 81) = 0.82	*F*(1, 81) = 0.53	*F*(2, 81) = 2.51	—
	F-ML	*F*(2, 81) = 0.77	*F*(1, 81) = 0.01	*F*(2, 81) = 1.55	—

CoP: center of pressure; Amp: amplitude (mm); F: frequency (Hz); AP: anteroposterior; ML: mediolateral; conditions: baseline and experimental conditions with the concurrent cognitive task; HC: healthy control; CFS: chronic fatigue syndrome; FM: fibromyalgia; ^*∗*^*p* < 0.05; ^*∗∗*^*p* < 0.010.

## Data Availability

The data used to support the findings of this study are available from the corresponding author upon request.
